# Stability and Variability of Indoor Room Transitions and Mild Cognitive Impairment in Older Adults

**DOI:** 10.1093/geroni/igab046.1724

**Published:** 2021-12-17

**Authors:** Chao-Yi Wu, Christina Reynolds, Lisa Barnes, Lisa Silbert, Hiroko Dodge, Jeffrey Kaye, Zachary Beattie

**Affiliations:** 1 Oregon Health & Science University, Portland, Oregon, United States; 2 Rush University Medical Center, Chicago, Illinois, United States

## Abstract

Indoor room transition is an underexplored real-world activity outcome. We estimated the stability and variability of indoor room transitions and their associations with mild cognitive impairment (MCI) in older adults. Older adults living-alone (n=159, age=78.3±8.8 years, 14% MCI) from the Oregon Center for Aging & Technology (ORCATECH) and the Minority Aging Research Study (MARS) were included. Room transitions were detected using passive infrared motion sensors in bathroom, bedroom, kitchen, and living room. The hourly number of room transitions was used to calculate the interdaily stability and intradaily variability of room transitions. MCI was operationalized by the Clinical Dementia Rating equaled 0.5. Generalized estimating equations models adjusted for demographics, health, and environmental factors revealed that older adults with MCI had a lower interdaily stability of room transitions than cognitive healthy peers (z=-2.06,p=0.03). A pervasive-sensing system deployed in homes can obtrusively measure room transition activities to inform cognitive health in older adults.

